# APDL1-CART cells exhibit strong PD-L1-specific activity against leukemia cells

**DOI:** 10.18632/aging.202578

**Published:** 2021-02-26

**Authors:** Qunyi Peng, Xiongpeng Zhu, Chuntuan Li, Pengliang Xin, Yan Zheng, Shengquan Liu

**Affiliations:** 1 Department of Haematology, Quanzhou First Hospital of Affiliated to Fujian Medical University, Quanzhou 362000, China

**Keywords:** leukemia, PD-1/PD-L1, aPDL1-CART cells, MEDI4736, immune checkpoint inhibitor

## Abstract

Chimeric antigen receptor (CAR) T cells target specific tumor antigens and lyse tumor cells in an MHC-independent manner. However, the efficacy of CAR-T cell and other cancer immunotherapies is limited by the expression of immune-checkpoint molecules such as programmed death-ligand 1 (PD-L1) on tumor cells, which binds to PD-1 receptors on T cells leading to T cell inactivation and immune escape. Here, we incorporated a PD-L1-targeted single-chain variable fragment (scFv) fusion protein sequence into a CAR vector to generate human anti-PD-L1-CAR-T cells (aPDL1-CART cells) targeting the PD-L1 antigen. Unlike control T cells, aPDL1-CART cells significantly halted the expansion and reduced the viability of co-cultured leukemia cells (Raji, CD46, and K562) overexpressing PD-L1, and this effect was paralleled by increased secretion of IL-2 and IFN-γ. The antitumor efficacy of aPDL1-CART cells was also evaluated *in vivo* by co-injecting control T cells or aPDL1-CART cells along with PDL1-CA46 cells to generate subcutaneous xenografts in NCG mice. Whereas large tumors developed in mice inoculated with PDL1-CA46 cells alone or together with control T cells, no tumor formation was detected in xenografts containing aPDL1-CART cells. Our data suggest that immune checkpoint-targeted CAR-T cells may be useful for controlling and eradicating immune-refractory hematological malignancies.

## INTRODUCTION

Leukemia comprises a group of life-threatening, malignant disorders of the blood and bone marrow [1]. There were 437,003 new leukemia cases and 309,006 leukemia-related deaths reported worldwide in 2018 [2]. Alarmingly, the incidence of leukemia continues to rise, contributing significantly to the global burden of diseases [3]. The treatment of leukemia mainly depends on disease type, severity, and individual patient characteristics [4]. Presently, the primary options for the management of leukemia include chemotherapy, radiotherapy, biological (i.e. immune-based) therapy, targeted therapy, surgery, and stem cell transplantation [1].

Previous studies have shown that engineered T cells engrafted with antigen-specific receptors may induce major histocompatibility complex (MHC)-independent immune responses that efficiently enhance anti-tumor immunity and cytokine production [5–7]. Chimeric antigen receptors (CARs) are constructed by linking the variable regions of the heavy and light chains of an antibody specific for a tumor cell surface molecule to the intracellular activation domain of the T-cell receptor. This strategy has further been optimized in newer generations of CAR designs that include also the intracellular domains of costimulatory receptors [8–10]. CAR-grafted T-cells (CAR-T cells) have demonstrated great potential for tumor immunotherapy in a variety of human cancers, including hematologic malignancies [11–15]. Despite striking clinical successes, current CAR-T cell therapies are sometimes associated with adverse events that preclude broader therapeutic implementation. Such events include cytokine release syndrome (CRS), B-cell aplasia, and neurological toxicities, all of which have been reported in CAR-T cell-based treatments of hematologic malignancies [16, 17]. These complications have been shown to arise from off-target interactions between CAR-T cells and normal cells sharing the tumor-associated antigens against which the CAR-T cells are directed [18, 19].

Programmed death ligand 1 (PD-L1, also termed B7-H1 or CD274) is commonly expressed by tumor cells and binds to the programmed death 1 (PD-1) immune checkpoint receptor on T cells. This interaction leads to T cell inactivation and exhaustion, allowing tumor cells to escape T-cell mediated destruction [20]. Accordingly, PD-L1 expression has been shown to correlate with rapid progression and poor outcomes in several cancer types [21]. Among hematological malignancies, PD-L1 expression is characteristically high in classical Hodgkin lymphoma, where clinical success has been achieved with PD-1/PD-L1 inhibitors [22]. Although the expression and functional influence of the PD-1/PD-L1 axis in leukemic variants remain less certain, aberrant PD-1/PD-L1 expression was shown to contribute to T cell dysfunction in chronic lymphocytic leukemia [23]. In addition, PD-L1 expression was recently reported as a negative prognostic variable in leukemia patients carrying NPM1 and FLT3 mutations [24].

Previous studies have shown that CAR-T cell inactivation can be elicited by PD-1/PD-L1 interaction and prevented instead by PD-1 or PD-L1blockade [25]. Diverse inhibitors of immune checkpoint signaling molecules (i.e. PD-1, PD-L1, and CTLA-4) have demonstrated impressive clinical activities against a variety of tumors [26–28]. However, the clinical application of immune checkpoint blockade therapy is still restricted by a low response rate and high systemic toxicity [29–31]. To address the current limitations associated with the use of immune checkpoint inhibitors available for cancer therapy [32], we incorporated a PD-L1-specific targeting sequence into a CAR construct to generate human aPDL1-CAR-T cells. Our findings provide evidence that this strategy remarkably increases CAR-T cell activity against PDL1-positive leukemia cells, and opens the door to further research to confirm and validate its clinical applicability.

## RESULTS

### Design and characterization of PDL1-K562 cells

**Figure 1:**
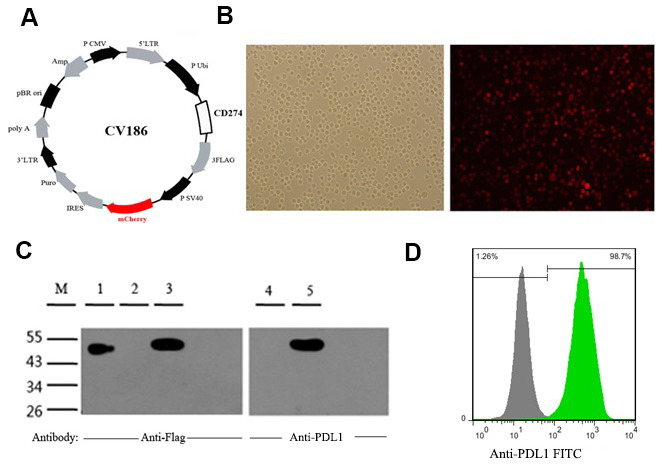
**Generation and validation of PDL1-K562 cells.** (**A**) Schematic representation of the lentiviral vector used to overexpress the human CD274 gene (GenBank accession number: NM_014143). (**B**) Detection of mCherry fluorescent protein expression in PDL1-K562 cells by fluorescence microscopy (100×). (**C**) Western blotting detection of PD-L1 in K562 cells. A 48-55 kDa protein band consistent with glycosylated PD-L1 was identified. Lanes 1-3, anti-flag antibody; Lanes 4-5, anti-PDL1 antibody; Lane 1, SURVIVIN-3FLAG-EGFP (48 kDa); Lanes 2 and 4, K562 cell lysates; Lanes 3 and 5, PDL1-K562 cell lysates. (**D**) Flow cytometry data showing that PD-L1 is expressed on the surface of almost all PDL1-K562 cells.

Co-expression of the CD274 gene, encoding PD-L1, and mCherry fluorescent protein was achieved in human leukemia K562 cells by lentiviral transduction (Figure 1A, 1B). Consistent with a previous report [33], a 48-55 kDa protein band corresponding to fully glycosylated PD-L1 was detected by western blotting in PDL1-K562 cells (Figure 1C). Flow cytometry further showed that PD-L1 was expressed on the surface of almost all PDL1-K562 cells (98.27% ± 0.15%; Figure 1D).

### aPDL1-CAR construction and generation of aPDL1-CART cells

**Figure 2:**
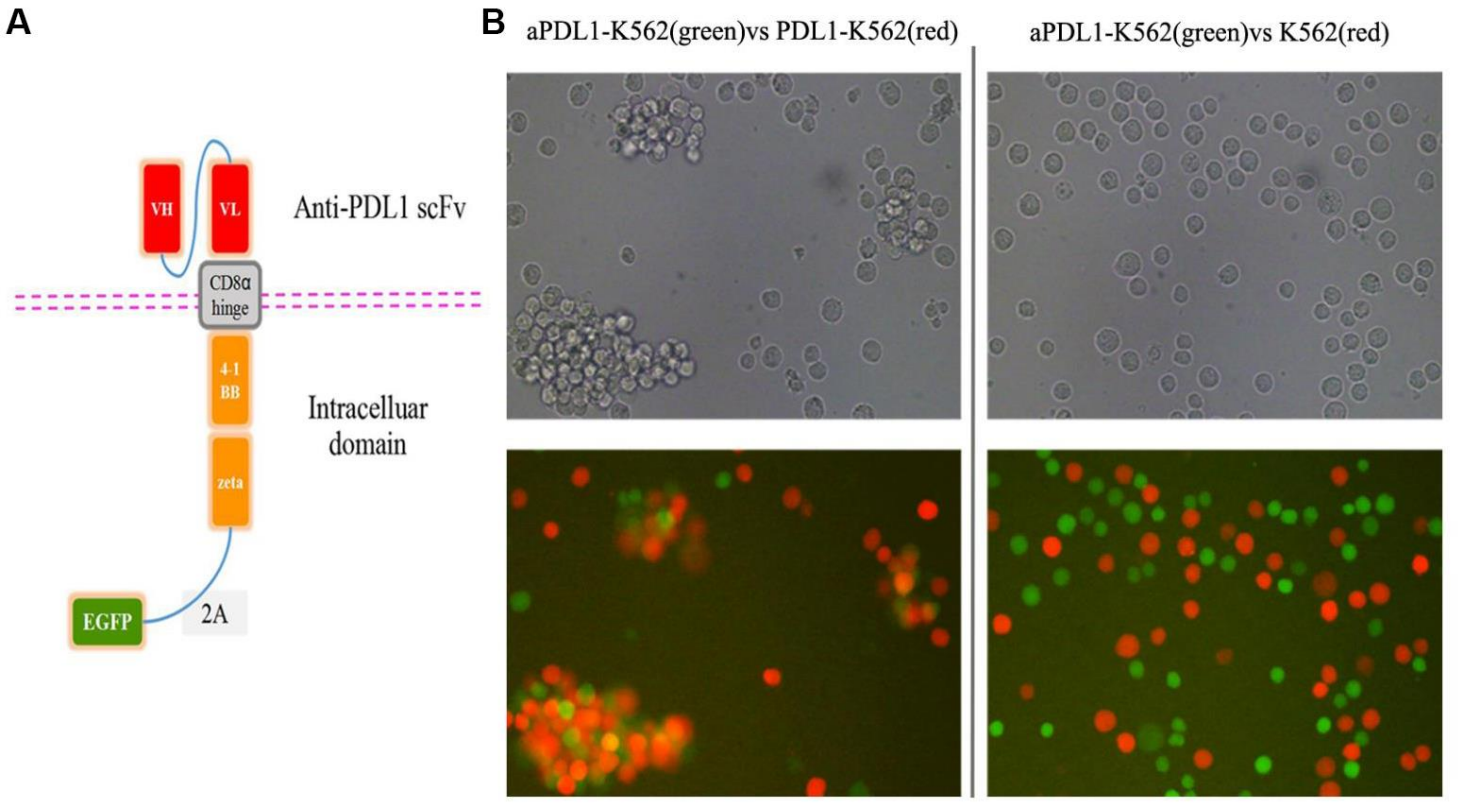
**Design and *in vitro* cell testing of the aPDL1-CAR construct.** (**A**) Structure of the aPDL1-CAR vector. The extracellular binding region consists of an anti-PDL1 scFv, the hinge and transmembrane domains correspond to CD8α, and the intracellular portion contains the signaling domains of 4-1BB and CD3ζ. The EGFP gene is linked by a 2A peptide sequence. (**B**) Transmitted light and fluorescence microscopy images of aPDL1-K562 cells in co-culture with PDL1-K562 cells or K562 cells for 4 h (200×). Green labeling corresponds to aPDL1-K562 cells, and red labeling indicates PDL1-K562 and K562 cells.

A CAR targeting PD-L1 was constructed by linking the single-chain variable fragment (scFv) sequence of the humanized anti-PD-L1 monoclonal antibody MEDI4736 to the hinge and the transmembrane domains of CD8α and the intracellular domains of CD3ζ and 4-1BB [34, 35]. To verify transduction efficiency, the EGFP gene was added to the construct (Figure 2A). To assess the binding ability of the scFv domain, K562 cells were transduced with the aPDL1-CAR construct to generate aPDL1-K562 cells. Following 4-h co-culture of PDL1-K562 cells and aPDL1-K562 cells or control K562 cells, microscopic examination revealed that aPDL1-K562 cells tended to aggregate around and bind PDL1-K562 cells more obviously than did control K562 cells (Figure 2B). These data indicate that aPDL1-K562 cells can target cells expressing PD-L1.

**Figure 3:**
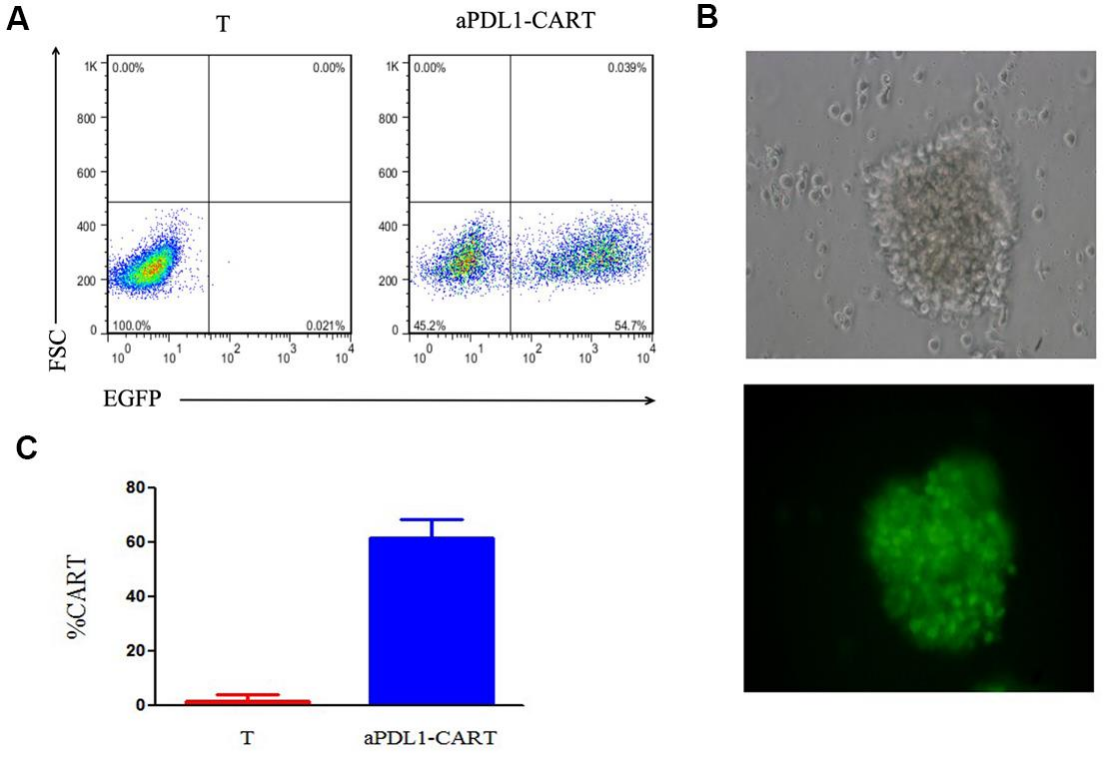
**Assessment of aPDL1-CAR transduction efficiency.** (**A**) Identification of aPDL1-CART cells by flow cytometry analysis of EGFP fluorescence (right panel); non-transduced T cells served as control (left panel). (**B**) Fluorescence microscopy of aPDL1-CART cells expressing EGFP (green fluorescence) (200×). (**C**) Quantitation of aPDL1-CAR vector transduction efficiency. * *P* < 0.05.

To examine the ability of PD-L1-targeted human T cells to lyse PD-L1-expressing leukemia cells, aPDL1-CART cells were generated using T cells isolated from healthy volunteers. aPDL1-CAR expression in these cells was confirmed by EGFP fluorescence 8 days post-transduction via flow cytometry (Figure 3A). Fluorescence microscopy further confirmed EGFP expression in aPDL1-CART cells (Figure 3B), and transduction efficiency was estimated to be 61.85 ± 6.51% (Figure 3C).

### aPDL1-CART cells possess PD-L1-specific activity and release IL-2 and IFN-γ

**Figure 4:**
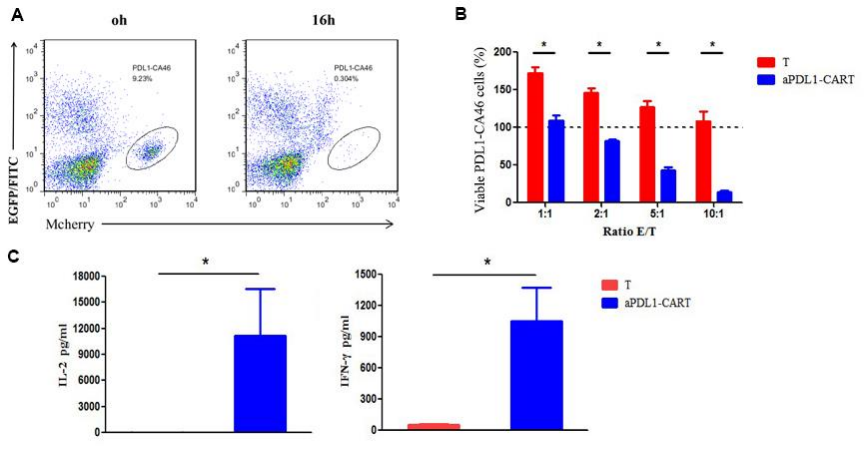
**Cytotoxicity and cytokine secretion analyses on aPDL1-CART cells *in vitro*.** (**A**) The cytotoxic activity of effectors was evaluated by flow cytometry based on distinct labeling of target cells (mCherry+) and aPDL1-CART cells (EGFP+) following co-culture with PDL1-CA46 cells. (**B**) Cytotoxic effects of control T cells (red) and aPDL1-CART cells (blue) against PDL1-CA46 cells at different E:T ratios. * *P* < 0.05. (**C**) ELISA measurements of IL-2 and IFN-γ secretion in control T cells (red) and aPDL1-CART cells (blue). * *P* < 0.05.

The cytotoxic activity of effectors was evaluated by flow cytometry based on distinct labeling of target cells (mCherry+) and aPDL1-CART cells (EGFP+) (Figure 4A). The viability of PDL1-CA46 cells, based on the initial population density (100%), was not decreased upon 16-h co-culture with control T cells at any effector-to-target (E:T) ratio. Indeed, even at lower E:T ratios, significant proliferation of PDL1-CA46 cells was observed (Figure 4B). In contrast, following co-culture with aPDL1-CART cells, a significant reduction in PDL1-CA46 cell expansion was detected at all tested E:T ratios (*P* < 0.05). At the lowest E:T ratio of 1:1, the total PDL1-CA46 cell population was reduced from 171.92% in the presence of control T cells to 108.78% upon co-culture with aPDL1-CART cells. In turn, a dramatic decrease in PDL1-CA46 cell viability occurred at higher E:T ratios. For instance, at an E:T ratio of 10:1, viable PDL1-CA46 cells constituted only 13.72% of the original population (Figure 4B). These findings demonstrate that aPDL1-CART cells exhibit cell dose-dependent cytotoxicity against PDL1-CA46 cells.

Next, we complemented the above experiments by comparing IL-2 and IFN-γ secretion between control T cells and aPDL1-CART cells *in vitro* using ELISA. As shown in Figure 4C, aPDL1-CART cells released significantly more IL-2 into the culture media than control T cells (11,144.74 vs. 19.07 pg/ml, respectively; *P* < 0.05). As expected, aPDL1-CART cells secreted also higher amounts of IFN-γ relative to control T cells (1,053.22 vs. 53.98 pg/ml, respectively; *P* < 0.05). These data indicate that aPDL1-CART cells show enhanced IL-2 and IFN-γ production.

### aPDL1-CART cells display PD-L1-specific activity against leukemia cells

**Figure 5:**
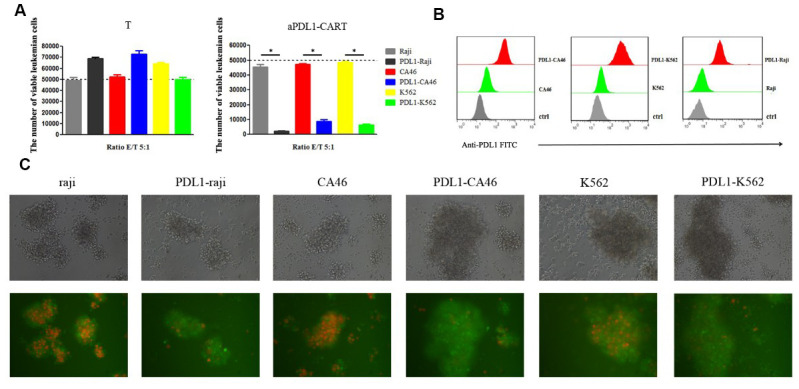
**aPDL1-CART cells display PDL1-specific activity against PD-L1-expressing leukemia cells.** (**A**) Analysis of the cytotoxicity of control T cells and aPDL1-CART cells against control and PD-L1-expressing leukemia cell lines following co-culture for 24 h; * *P* < 0.05. (**B**) Flow cytometry detection of PD-L1 expression in PD-L1-transduced and control leukemia cells. (**C**) Transmitted light and fluorescence microscopy imaging of aPDL1-CART cells co-cultured with leukemia cells for 24 h (200×).

To confirm that aPDL1-CART cells elicit specific cytotoxicity against PD-L1-expressing leukemia cells, aPDL1-CART and control T cells were independently co-cultured, for 24 h and at an E:T ratio of 5:1, with Raji, CA46, and K562, PDL1-Raji, PDL1-CA46, and PDL1-K562 cells. Co-cultures with the corresponding non-PD-L1-transduced parental leukemia cells were used as control. Flow cytometry assays showed that the number of viable leukemia cells (either control or PD-L1-expressing cells) was not significantly decreased upon co-culture with T cells. Thus again suggested that naïve T cells can not eliminate leukemia cells efficiently. Likewise, 24-h co-culture with aPDL1-CART cells did not decrease the viability of parental Raji, CA46, and K562 cells, but instead it dramatically reduced the number of viable PDL1-Raji, PDL1-CA46, and PDL1-K562 cells (*P* < 0.05, Figure 5A). Successful transduction of PD-L1 in leukemia cells was confirmed by flow cytometry, which showed low PD-L1 expression in parental Raji, CA46, and K562 cells, and moderate to high PD-L1 expression in PDL1-Raji, PDL1-CA46, and PDL1-K562 cells (Figure 5B). These findings were confirmed by fluorescence microscopy, which displayed a small proportion of viable, mCherry-positive PD-L1-expressing leukemia cells but numerous viable control cells upon co-culturing with aPDL1-CART cells (Figure 5C). These data confirm that aPDL1-CART cells have specific antitumor activity against PD-L1-expressing leukemia cells.

### aPDL1-CART cells inhibit leukemia cell growth *in vivo*

**Figure 6:**
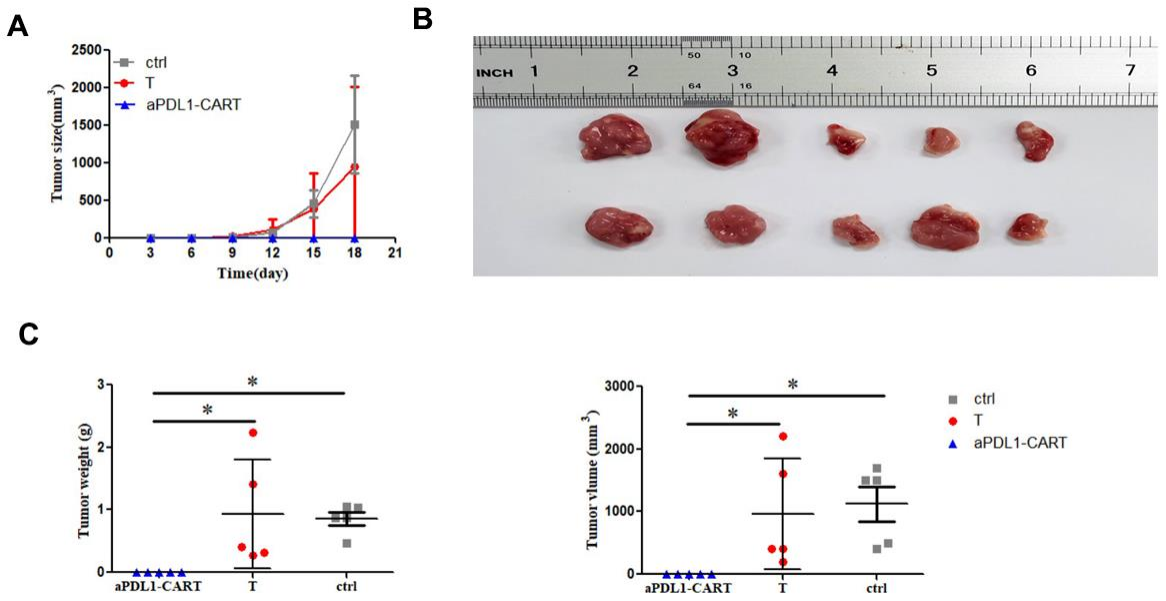
***In vivo* antitumor activity of aPDL1-CART cells.** (**A**) Growth curve of xenografted PDL1-CA46 cells. (**B**) Volume measurements of tumor xenografts formed by PDL1-CA46 cells either alone or after co-injection with control T cells or aPDL1-CART cells. (**C**) Comparison of tumor weight and volume for the three experimental groups. * *P* < 0.05.

Finally, we verified the antitumor activity of aPDL1-CART cells by generating tumor xenografts in NCG mice by subcutaneous injection of PDL1-CA46 cells, either alone or together with aPDL1-CART or control T cells. Tumor formation was observed by day 8 post-injection in mice injected with PDL1-CA46 cells alone or together with control T cells. By day 18 post-injection, the corresponding tumors reached 1,800 mm^3^ and all mice were humanely sacrificed (Figure 6A). In contrast, no tumor development was observed in mice co-injected with aPDL1-CART cells (Figure 6A and 6B). Accordingly, significant differences in tumor weight and volume were recorded between tumors admixed with aPDL1-CART cells and those formed by PDL1-CA46 cells alone or containing control T cells (*P* < 0.05, Figure 6C). These data demonstrate that aPDL1-CART cells, but not naïve T cells, successfully prevent the development of PD-L1-expressing leukemia xenografts in immunocompromised mice.

## DISCUSSION

In the immunosuppressive tumor microenvironment, hypofunction of T cells often occurs in association with PD-L1 up-regulation in cancer cells [36]. The latter represents a critical obstacle preventing tumor eradication by cells of the immune system and has proved to significantly restrict the efficacy of cancer immunotherapies [27]. Binding of PD-L1 expressed on the surface of tumor cells to the PD-1 immune checkpoint receptor on T cells inhibits the response of activated T cells to tumor antigens and causes T cell exhaustion [26]. Therefore, immune-checkpoint blockade has emerged as a promising strategy for cancer therapy [37, 38].

In this study we tested the hypothesis that integrating specific antibody recognition sites for PD-L1 into a CAR will endow T cells with both specificity against PD-L1-expressing leukemia cells and resistance to PD-1/PD-L1-mediated immunosuppression. To this end, a new aPDL1-CART cell line was generated by fusing a human PD-L1-directed scFv sequence into a CAR construct containing the intracellular regions of T cells’ activating (CD3ζ) and co-stimulatory (4-1BB) signaling domains [34, 35]. We showed that aPDL1-CART cells specifically recognized and lysed leukemia cells expressing PD-L1 antigens in an MHC-independent manner. Our results are in line with recent research showing that the combination of CAR-T cells and an oncolytic adenovirus expressing a PD-L1 mini-antibody reduces the progression of solid tumors [39], as well as with studies reporting the feasibility of CAR-based approaches directly targeting the PD-L1/PD-1 pathway [40–42]. In the current study, we designed the CAR vector to contain the extracellular domain of an anti-PDL1 scFv derived from MEDI4736, an specific PD-L1 antagonistic monoclonal antibody that showed to potentiate T-cell mediated antitumor activity and improve the survival of tumor-bearing mice [34].

We obtained initial evidence of the feasibility of our approach by demonstrating that transduction of the aPDL1-CAR construct into K562 cells determined efficient binding to PDL1-expresing K562 cells. Of note, our initial attempts to optimize the generation of aPDL1-CART cells revealed that excessive viral vector loads led to apoptosis, while low viral inocula resulted in low aPDL1-CAR expression rates (data not shown). The best transduction conditions were finally achieved using a MOI of 10, which resulted in a transduction efficiency of 61.85 ± 6.51%.

Our cytotoxicity assays showed that upon co-culturing with activated, non-CAR transduced T cells, PD-L1-expressing leukemia cells continued proliferating, even at an E:T ratio of 1:1. However, LDH and chromium release experiment failed to discover this phenomenon (data not provided). The proliferation rate of tumor cells is a well-known factor decreasing the efficacy of immunotherapies. In contrast, at all the E:T ratios tested, the lytic activity of aPDL1-CART cells against PD-L1-expressing leukemia cells was markedly higher than that recorded for control T cells. Paralleling these findings, and as reported in other CART studies [43–45]. Cytokine expression assays indicated that aPDL1-CART cells secreted large amounts of IL-2 and IFN-γ compared to control T cells. We thus concluded that aPDL1-CART cells display potent *in vitro* activity against leukemia cells in a PD-L1-dependent manner. Finally, we tested the *in vivo* efficacy of aPDL1-CART cells against leukemia cells in a xenograft mouse model. Whereas all the mice injected with PDL1-CA46 leukemia cells with or without control T cells developed large tumors, no tumor growth was observed after co-injection of aPDL1-CART cells over an 18-day period. Since all mice were sacrificed at this time point, the potential development of tumors in the aPDL1-CART group after this time cannot be excluded. Nevertheless, our data demonstrate aPDL1-CART cells effectively prevented the growth of PDL1-expressing leukemia cells *in vivo*.

The present study has some limitations. First, the possibility exists that aPDL1-CART cells mediate autoimmunity against normal cells expressing PD-L1; however, we observed no gross evidence of the latter in the mice co-injected with aPDL1-CART cells. Second, the *in vivo* tumor model was established in NCG mice by subcutaneous injection of leukemia cells; however, intravenous injection or allowing the formation of a tumor first before the injection of the recombinant construct is a more favorable approach to establishing the tumor xenograft mouse model. Third, all mice were sacrificed 18 days post-injection due to ethical considerations, therefore the observation period was relatively short. Fourth, our *in vivo* experiments were performed in NCG mice, which lack functional T, B, and NK cells. Thus, the potential immunoregulatory influence of these cell types on both tumor growth and CART activity could not be examined. Further studies to overcome these shortages seem justified to validate the findings from this study.

In summary, the present study demonstrated that aPDL1-CART cells have strong PD-L1-specific activity against leukemia cells both *in vitro* and *in vivo*. This evidence suggests that integrating functional immune-checkpoint blocking domains into CARs may be a valuable approach to overcome tumor immunosuppression and improve CAR T-cell therapies.

## MATERIALS AND METHODS

### Ethical approval

The protocol for human studies and cell experiments was reviewed and approved by the Ethical Review Committee of The First Hospital of Quanzhou Affiliated to Fujian Medical University (approval No. 2018121). All human studies were conducted following the Declaration of Helsinki, and written informed consent was obtained from 3 healthy volunteers following a detailed description of the purpose of the study. All animal experiments were performed in accordance with the National Regulations for the Care and Use of Laboratory Animals (2017 revision) issued by the State Council of China, and were approved by the Ethics Review Committee on Laboratory Animals of Huaqiao University (approval No. A2019043).

### Animals

Four- to six-week-old mice of the NCG strain (NOD/ShiLtJGpt-Prkdcem26Cd52Il2rgem26Cd22/Gpt) were purchased from GemPharmatech Co., Ltd. (Nanjing, China). Mice were housed in a specific pathogen-free facility for a week prior to experiments and had free access to food and water.

### Construction of the aPDL1-CAR

A single-chain antibody fragment (scFv) sequence comprising the variable regions of the heavy and light chains of MEDI4736, a humanized anti-PD-L1 monoclonal antibody, was fused to the hinge and the transmembrane domains of CD8α and the intracellular domains of 4-1BB and CD3ζ [8, 34, 35]. To monitor gene-transfer efficiency, the resulting aPDL1-CAR construct was linked to the enhanced green fluorescent protein (EGFP) gene by a 2A peptide sequence. The aPDL1-CAR construct was synthesized by Shanghai GeneChem Co., Ltd. (Cat. No. GORL0177591; Shanghai, China) and confirmed by sequencing.

### Cell lines and culture

Human erythroleukemia K562 cells were a gift from Fujian Institute of Hematology (Fuzhou, China). The CD274 gene was lentivirally transduced into K562 cells to produce PDL1-K562 cells expressing PD-L1 and mCherry fluorescent protein. These cells were also transduced with a CV186 empty control vector without CD274 gene to stably express mCherry fluorescent protein alone. In addition, the aPDL1-CAR construct was separately transduced to generate aPDL1-K562 cells.

Human Burkitt’s lymphoma Raji cell line (Cat. No. KGB500) was purchased from Jiangsu KeyGEN BioTECH Co., Ltd. (Nanjing, China). Human Burkitt’s lymphoma CA46 cells were obtained from Fujian Institute of Hematology (Fuzhou, China). The CD274 gene (GenBank accession No. NM_014143) was transduced into Raji and CA46 cells using a CV186 lentiviral vector to produce PDL1-Raji and PDL1-CA46 cells expressing PD-L1 and mCherry fluorescent protein (Figure 1A). Both cell lines were also engineered to permanently express the mCherry fluorescent protein alone. All cells were cultured in RPMI 1640 medium (Gibco; Grand Island, NY, USA) supplemented with 10% fetal bovine serum, 100 UI/ml penicillin, and 100 μg/ml streptomycin (HyClone; Logan, UT, USA). Cells were maintained in a humidified atmosphere containing 5% CO_2_ at 37° C.

### Co-culture assay

To assess the binding ability of the scFv domain to PD-L1-expressing cells, 1 × 10^5^ aPDL1-K562 cells were co-cultured with PDL1-K562 or K562 cells at a ratio of 1:1 for 4 h. Cell interactions were visualized by fluorescence microscopy (Nikon TE2000-U; Nikon Corporation; Tokyo, Japan).

### T cell isolation, culture, and aPDL1-CAR transduction

Whole blood samples were collected from 3 healthy volunteers, and peripheral blood mononuclear cells (PBMCs) were prepared using Ficoll density gradient centrifugation. Cells were activated in a plate coated with anti-CD3 monoclonal (OKT3; 5 μg/ml; Takara, Kyoto, Japan) and anti-CD28 monoclonal (1 μg/ml; eBioscience, San Diego, CA, USA) antibodies. T cells were incubated in RPMI 1640 medium supplemented with IL-2 (200 U/ml; Jiangsu Sihuan Bioengineering Co., Ltd., Jiangyin, China) at a density of 1 × 10^6^ cells/ml. Two days later, T cells were transduced with lentiviral vectors encoding the aPDL1-CAR construct (titer: 2 × 10^8^ TU/ml; Shanghai GeneChem Co., Ltd., Shanghai, China). To this end, activated T cells were re-suspended at a density of 1 × 10^6^ cells/ml and seeded with IL-2 (30 IU /ml) onto 24-well plates coated with RetroNectin (15 μg/ml; Takara, Kyoto, Japan). aPDL1-CAR lentiviral vectors were added into cell cultures at a multiplicity of infection (MOI) of 10 and centrifuged at 1,000 g for 1 h. The medium was substituted after 48 h, and cells were then maintained in RPMI 1640 supplemented with IL-2 (200 U/ml). Transduction efficiency was evaluated by flow cytometry at day 8 post-transduction.

### Cytotoxicity assay

The cytotoxic activity of T cells and aPDL1-CART cells was evaluated by flow cytometry based on distinct labeling of target cells (mCherry+) and aPDL1-CART cells (EGFP+). Briefly, 5 × 10^4^ mCherry-labeled PDL1-CA46 cells were co-cultured with effector cells at various effector-to-target (E:T) ratios for 16 h. Then, total cell number in each cell mixture was determined with a JIMBIO-FIL cell counter (Jiangsu Jimbio Technology Co., Ltd.; Changzhou, China). Total cell populations (i.e. including dead cells) were stained with Annexin V-FITC staining solution (Cat. No. 556420; BD Biosciences, Bedford, MA, USA) and the percentage of intact (Annexin-V-FITC negative and mCherry positive) leukemia was determined by flow cytometry. Cytotoxicity was then assessed by flow cytometry by quantification of viable (Annexin-V-FITC negative and mCherry positive) leukemia cells using the following formula: viable leukemia cells (%) = [(total number of mixed cells × percentage of intact leukemia cells)/initial number of leukemia cells] × 100%. All assays were repeated 3 times.

To evaluate cytolytic activity against leukemia cells, T cells and aPDL1-CART cells were co-cultured with 5 × 10^4^ leukemia cells (Raji, CA46, K562, PDL1-Raji, PDL1-CA46, or PDL1-K562 cells) at an E:T ratio of 5:1 for 24 h. Cell interactions were visualized by fluorescence microscopy. Cytolytic activity was then evaluated by computing the number of viable (Annexin-V-FITC negative and mCherry positive) cells using the formula: number of viable leukemia cells = total number of mixed cells × percentage of intact leukemia cells. All assays were repeated 3 times.

### Cytokine detection

aPDL1-CART cells and T cells were independently co-cultured with PDL1-CA46 cells (5 × 10^4^) in 24-well plates at an E/T ratio of 10:1 for 24 h. Then culture supernatants were harvested and IL-2 and IFN-γ secretion was quantified with ELISA kits (Shanghai Excell Biological Technology Co., Ltd.; Shanghai, China) following the manufacturer’s protocol. All data were analyzed with i-control software version 1.9 (Tecan's Infinite® microplate series; Männedorf, Switzerland).

### PD-L1 expression assays

PD-L1 expression was determined using western blotting and flow cytometry. Briefly, 5 × 10^6^ cells were harvested and lysed in RIPA buffer, and protein concentration was quantified using an Enhanced BCA Protein Assay kit (Beyotime Biotechnology; Shanghai, China). Then, 20 μg of total protein per sample was separated by SDS-PAGE and transferred onto PVDF membranes. Following membrane blocking with 5% nonfat dry milk in TBS wash buffer with Tween-20 (TBS-T) at 4° C overnight, immunoblots were probed with anti-Flag (1:100 dilution; Cat No. 2368S; Cell Signaling Technology, Danvers, MA, USA) and anti-PDL1 antibodies (1:100 dilution, Cat. No. ab205921; Abcam, Cambridge, UK). Then, the immunoblots were incubated with an HRP-linked secondary antibody (1:1000; Cat. No. 7074S; Cell Signaling Technology) at 37° C for 2 h and washed with TBS-T. Protein bands were visualized with a Molecular Imager VersaDoc MP 4000 system (Bio-Rad; Hercules, CA, USA).

PD-L1 expression was also detected using flow cytometry. Briefly, cells were incubated with a FITC-conjugated mouse anti-human CD274 antibody (Cat. No. 558065; BD Biosciences). Flow cytometry was used to ascertain PD-L1 expression in PD-L1-transduced and control leukemia cells. All flow cytometry experiments were performed on a FACS Calibur Flow Cytometer (BD Biosciences) and the resulting data analyzed with FlowJo software version 7.0.

### NCG mouse xenograft tumor model

Animals were subcutaneously injected with PDL1-CA46 cells (0.5 × 10^6^ cells per mouse), either alone or together with effector T cells at an E:T ratio of 5:1. Mice co-injected with aPDL1-CART cells or T cells constituted the experimental groups, while mice injected with PDL1-CA46 cells alone served as controls. Tumor length and width were measured with a caliper every 3 days. Tumor volume was calculated using the following formula: tumor volume (mm^3^) = (tumor length x width^2^)/2. Mice were euthanized whenever tumor volume exceeded 1,800 mm^3^.

### Statistical analysis

Data analysis was performed with GraphPad Prism version 5 (GraphPad Software, Inc.; La Jolla, CA, USA). Student’s *t*-test was employed to compare the means between two groups, and Mann-Whitney *U* test was used to compare tumor xenograft weight and volume between groups. *P* < 0.05 was considered significant.
